# Mir-139-5p inhibits glioma cell proliferation and progression by targeting GABRA1

**DOI:** 10.1186/s12967-021-02880-9

**Published:** 2021-05-17

**Authors:** Lei Wang, Yan Liu, Zhengtao Yu, Jianwu Gong, Zhiyong Deng, Nianjun Ren, Zhe Zhong, Hao Cai, Zhi Tang, Haofeng Cheng, Shuai Chen, Zhengwen He

**Affiliations:** 1 Department of Neurosurgery, Hunan Cancer Hospital and The Affiliated Cancer Hospital of Xiangya School of Medicine, Central South University, No.283 Tongzipo road, Yuelu district, Changsha, 410006 Hunan China; 2 Department of Neurology, Changsha Central Hospital, University of South China, No.161 Shaoshan road, Yuhua district, Changsha, 410007 Hunan China; 3 Department of Neurosurgery, Haikou People’s Hospital, The Affiliated Haikou Hospital of Xiangya School of Central South University, No.43 Renmin road, Meilan district, Haikou, 570208 Hainan China

**Keywords:** Glioma, MiR-139-5p, GABRA1, Biomarker, Invasion, Migration, Proliferation, Apoptosis

## Abstract

Glioma is an extremely aggressive malignant neoplasm of the central nervous system. MicroRNA (miRNA) are known to bind to specific target mRNA to regulate post-transcriptional gene expression and are, therefore, currently regarded as promising biomarkers for glioma diagnosis and prognosis. The aim of the present study was to examine the pathogenesis and potential molecular markers of glioma by comparing the differential expression of miRNA and mRNA between glioma tissue and peritumor brain tissue. We explored the impact of screened core miRNA and mRNA on cell proliferation, invasion, and migration of glioma. An miRNA expression profile dataset (GSE90603) and a transcriptome profile dataset (GSE90598) were downloaded from combined miRNA-mRNA microarray chips in the Gene Expression Omnibus (GEO) database. Overall, 59 differentially expressed miRNAs (DEMs) and 419 differentially expressed genes (DEGs) were identified using the R limma software package. FunRich software was used to predict DEM target genes and miRNA-gene pairs, and Perl software was used to find overlapping genes between DEGs and DEM target genes. There were 129 overlapping genes regulated by nine miRNAs between target genes of the DEMs and DEGs. The Chinese Glioma Genome Atlas(CGGA) was analyzed in order to identify miRNAs with diagnostic and prognostic significance. MiR-139-5p, miR-137, and miR-338-3p were validated to be significantly linked to prognosis in glioma patients. Finally, we validated that miR-139-5p affected glioma malignant biological behavior via targeting gamma-aminobutyric acid A receptor alpha 1(GABRA1) through rescue experiments. Low miR-139-5p expression was correlated with survival probability and World Health Organization (WHO) grade. MiR-139-5p overexpression inhibited cell proliferation, migration, and invasion of glioma in vitro. GABRA1 was identified as a functional downstream target of miR-139-5p. Decreased GABRA1 expression was related to similar biological roles as miR-139-5p overexpression while upregulation of GABRA1 effectively reversed the inhibition effects of miR-139-5p. These results demonstrate a novel axis for miR-139-5p/GABRA1 in glioma progression and provide potential prognostic predictors and therapeutic target for glioma patients.

**Supplementary Information:**

The online version contains supplementary material available at 10.1186/s12967-021-02880-9.

## Introduction

Glioma is the most prevalent primary malignant neoplasm in the central nervous system, with high recurrence and mortality rates. According to the World Health Organization (WHO) grading system, glioma has been classified into four grades [1]. The higher the grade of the glioma, the worse the prognosis of the patient. Glioblastoma multiforme(GBM), represents the most aggressive glioma subtype, with a five-year survival rate of less than 3% and average survival of less than 12 months [2]. Treatment involves surgical resection followed by adjuvant chemotherapy or concurrent chemoradiotherapy [3]. However, the 3-year survival rate remains at a low level, especially for GBM patients, with the 3-year survival rate only slightly increasing from 2.00%–5.00% to 7.31% [4]. Therefore, researchers have turned their attention to study the potential molecular mechanisms of GBM, and to find novel prognostic biomarkers for the early diagnosis and monitoring of tumorigenesis and for evaluating prognosis.

MicroRNAs (miRNA) are commonly defined as non-coding RNAs consisting of 18–25 nucleotides that regulate post-transcriptional gene expression via specific binding to target mRNA. MiRNA are known to be involved in the regulation of tumor cellular physiological processes, and the aberrant expression and cross-regulation of miRNA and target mRNA can be applied as a potential diagnostic and prognostic biomarker in all grades of glioma. Researchers have begun identifying miRNAs that may act as glioma oncogenes or suppressor genes, which could then be applied as diagnostic and predictive biomarkers, and even act as molecular therapeutic targets against glioma [5, 6].

**Fig. 1:**
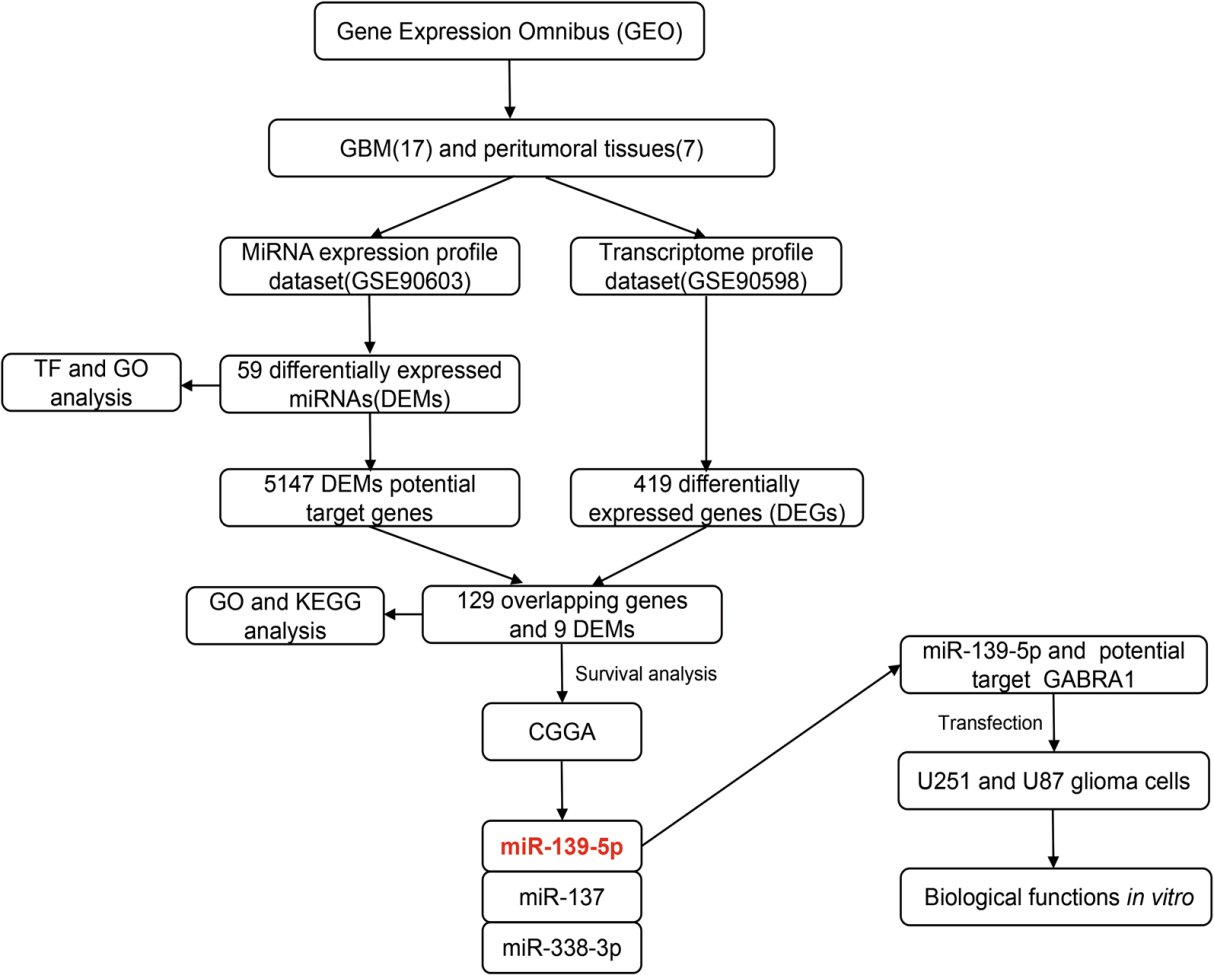
This figure presents an overview of the analysis workfow

To identify the novel diagnostic and predictive value of miRNAs as biomarkers, bioinformatics tools were used to integrate analysis of differential expression of miRNAs and mRNAs from paired 16 fresh-frozen GBM samples and seven peritumoral tissues. Via integrating the miRNA and mRNA expression in the GBM pairs, we identified a number of predictive miRNAs that can be used as biomarkers for diagnosis and prognosis of GBM, and which could then be used as potential clinical therapeutic targets (flow chart see Fig. 1). Specifically, we identified that miR-139-5p and GABRA1 served as inverse agents in the regulation of malignant phenotypes of glioma cells, we doubted whether the function of miR-139-5p on glioma cells was mediated through its inhibitory impression on GABRA1 expression. We, performed rescue experiments to dispose it. In this study, we firstly provide a series of potential targets for future investigation into the molecular mechanisms involved in glioma and offer insight into miRNAs as biomarkers for disease progression. Furthermore, we explore the possible role of miR-139-5p and GABRA1 in the development of glioma and the underlying molecular regulation mechanism. Our findings may provide new insights for the diagnosis and treatment of glioma.

## Methods

### Microarray data

GSE90603 and GSE90598 were downloaded from the GEO database. A total of 23 fresh-frozen samples were analyzed, including 16 individual samples of GBM and seven individual samples of healthy brain tissues. The expression of miRNA (GSE90603) was detected using the platform GPL21582 (Affymetrix Multispecies miRNA-4 Array). The mRNA dataset(GSE90598) was analyzed by using the platform GPL17692 (Affymetrix Human Gene 2.1 ST Array).

### Identification of DEMs and DEGs

The DEMs and DEGs between GBM samples and healthy brain tissues was processed using R software. The Affy R package was used to process the downloaded raw data and the limma R package was then used to identify DEMs and DEGs. The adjusted *p* values were used to decrease the false positive rate using Benjamini and Hochberg false discovery rate method by default. |log2FC|≥ 2 and adjust *p* < 0.05 were identified as the DEMs or DEGs between GBM sample and healthy brain tissues.

### Gene, function and pathway enrichment analysis

FunRich is a stand-alone software tool used mainly for functional enrichment and interaction network analysis of genes and proteins (http://www.funrich.org) [7]. FunRich was used to perform DEM downstream transcription factor analysis and DEM target genes prediction. In addition, GO function and KEGG pathway were analyzed using the FunRich software. The core miRNAs and their target genes regulatory network was constructed and visualized by Cytoscape 3.6.1 software.

### Survival analysis and glioma grading analysis

The Chinese Glioma Genome Altas (CGGA) database is a user-friendly web application for data storage and analysis to explore brain tumors datasets over 2,000 samples from Chinese cohorts. This database includes miRNA microarray (198 samples) and matched clinical data and could used for assessing the effect of certain miRNA on glioma patients survival. In addition, the miRNA expression level between grade II and grade IV glioma was analyzed via CGGA database. The median expression values of every DEMs in glioma samples were calculated, based on which they were divided into high (above median)-and low-expression(below median) groups. Integrated with the prognostic outcomes, including the overall survival, obtained from CGGA database, the Kaplan–Meier survival curves of these two groups were plotted and the log-rank tests were used to assess the relationship of gene expression to survival. A *p*-value < 0.05 was set as the threshold for statistical significance.

### Cell culture

Human brain glioma cell lines U251 and U87 were procured from BeNa Culture Collection (BNCC, China). Cell lines were cultivated in Dulbecco’s Modified Eagle’s Medium (DMEM, Sigma, USA) with 10% foetal bovine serum (FBS, Gibco, USA) in 5% CO_2_ at 37℃.

### Cell transfection

Lipofectamine 2000 (LIP2000, invitrogen, USA) was applied to transiently transfect the miR-139-5p mimics or inhibitor as well as the GABRA1 overexpression vector or GABRA1 siRNA into glioma cells according to the manufacture’s instruction. MiR-139-5p mimic, miRNA mimic negative control (mimic NC), miR-139-5p inhibitor control, miRNA inhibitor negative control (inhibitor NC) were chemically synthesized by HonorGene (China). GABRA1 overexpression plasmid and siRNA targeting GABRA1 were purchased from RiboBio (China).

### Quantitative Real time-PCR (qRT-PCR)

TRIzol Reagent (Thermo, USA) was applied to extract the total RNA from samples according to the manufacture’s recommendation. The reverse transcription of 200 ng total RNA was utilizing SuperRT RT reagent Kit (CWBio, China) and quantitative real-time PCR with SYBR PCR Master Mix(CWBio, China). The PCR was set at the initial denaturation of 10 min at 95℃, following with 15 s at 95℃, and 30 s at 60 °C in a total of 40 cycles. All experiments were carried out in triplicate. The miR-139-5p expression was normalized to U6 while the GABRA1 was normalized to actin. The relative expression ratios of genes were calculated by the 2-ΔΔCT method. The primers involved in this assay were shown as follow:

hsa-miR-139-5p: TCTACAGTGCACGTGTCTCC; U6, F: CTCGCTTCGGCAGCACA, R:AACGCTTCACGAATTTGCGT; actin, F:ACCCTGAAGTACCCCATCGAG, R:AGCACAGCCTGGATAGCAAC; GABRA1, F:ATGATGGAGCTCGAGGCAAA, R:AGCTCTGAATTGTGCTGGGT.

### Western blot assay

Total protein in cells was collected, and the concentration of protein wasquantified with Pierce BCA Protein Assay Kit. After segregated by 10% sodium dodecyl sulphate–polyacrylamide gel electrophoresis (SDS-PAGE) gel, extracted proteins were transferred to polyvinylidene difluoride (PVDF) membranes and blocked in Tris-buffered saline-Tween with 5% skim milk at room temperature for 60 min. Western blot analysis was performed according to standard procedures. Primary antibodies were GABRA1 (1:1000, proteintech, USA), β-actin (1:5000, proteintech, USA), and corresponding secondary antibodies were anti-mouse and anti-rabbit(proteintech, USA). X-ray film was pressed in dark. Finally, the proteins were detected by enhanced chemiluminescence (ECL), followed by expose about 60 s for scanning and measuring.

### EdU assay

The transfected glioma cells were seeded onto the six-well tissue-culturing plates (1 × 10^5^cells per well). Following the indicated treatments an EdU Apollo-567 assay kit (RiboBio, China) was utilized to test cell proliferation, with nuclear EdU and DAPI staining visualized under a fluorescent microscope.

### Cell apoptosis assay by flow cytometry

Apoptotic cells were tested by apoptosis detection kit (KeyGen BioTECH, China) according to the manufacturer's instructions. Cells under log phase were obtained. 500 μl binding buffer was used to resuspend cells after cells were collected. Cells were added with isometric 5 μl Annexin V-APC and 5 μl propidium iodide and incubated for 10 min at room temperature in the dark. Cells were tested using the flow cytometry.

### Wound-healing assay

At 6 h post-transfection, the cells were digested, centrifuged and re-suspended in FBS-free culture. The concentration of cells was adjusted to 5 × 10^5^ cells/ml. Then cell layers were scratched with a 100 μl sterile pipette tip. After removing cell culture medium and suspension cells and cell debris, each well was added with serum-free medium and stored in incubator for 24 and 48 h. Cell migration area was then viewed and photographed after incubation for 24 h. Meanwhile, scratch test was performed to evaluate the difference in cell healing ability according to the migration area.

### Transwell assay

The Matrigel (BD, USA) melted at 4℃ overnight. 100 μl of diluted matrigel was then added in the chamber. Afterwards, 200 μl of serum-free medium was added to the upper chamber; meanwhile, 500 μl of 10% FBS DMEM was added to the lower chambers, and 2 × 10^5^ collected cells in total were planted in the upper ones and cultivated in the incubator for another 48 h. Subsequently, the invading chamber was taken out, and cells on the polycarbonate membrane were fixed with 4% paraformaldehydel, followed by staining with 0.1% crystal violet. Three random fields were selected, and invaded cells were counted under a microscope. The experiments were carried out in triplicate.

### Statistical analysis

All data are presented as mean ± SD of three independent experiments. Comparisons between the quantitative data were made using Student’s t test, with *p* < 0.05 considered statistically significant. Survival rate was delineated using Kaplan–Meier method. Statistical and graphical analyses were performed with the use of GraphPad Prism 5 software.

## Results

### Identification of differentially expressed miRNAs (DEMs) and differentially expressed genes (DEGs) in GBM

**Fig. 2:**
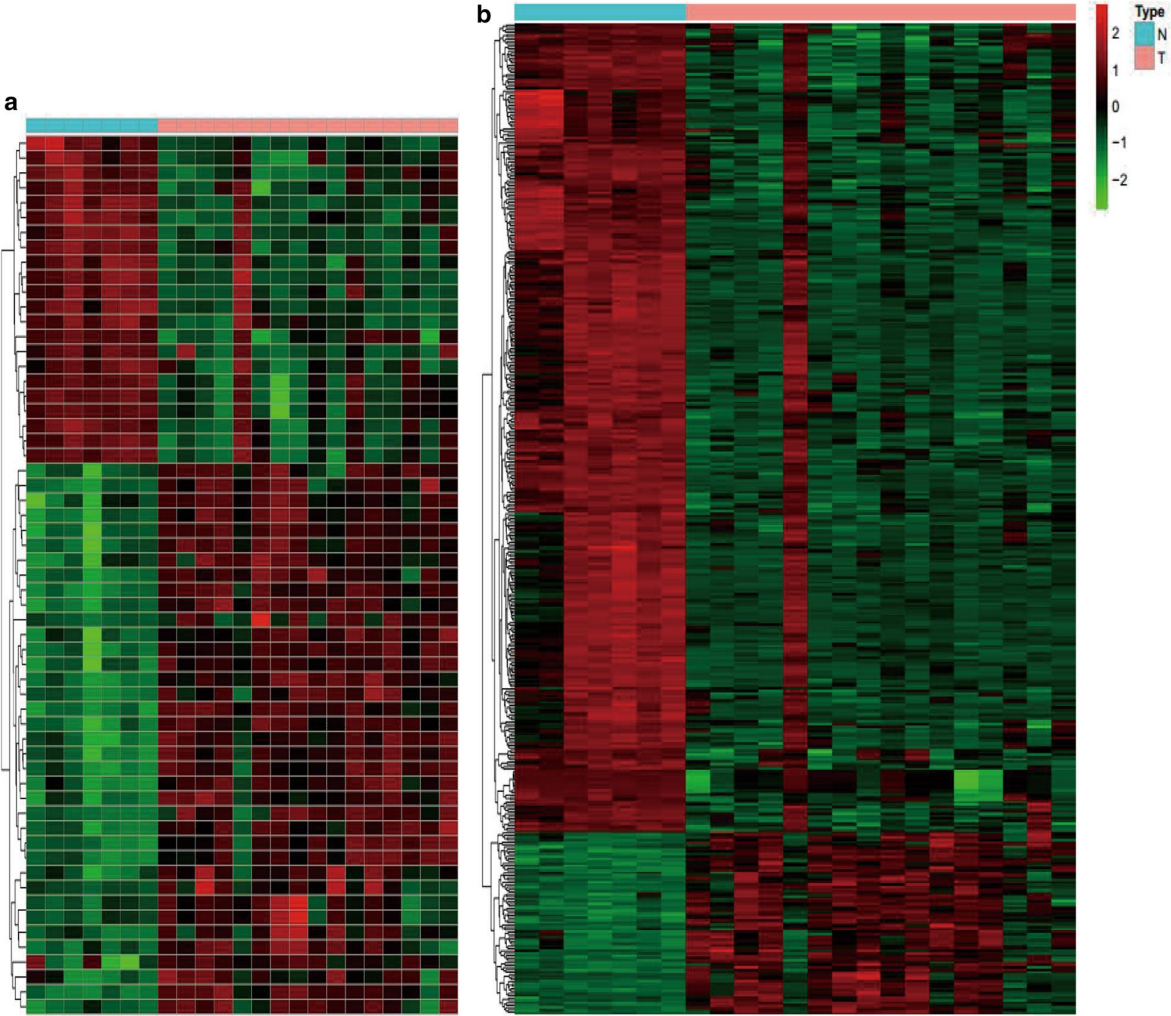
**a** Heat map revealing the expression profles of 59 DEMs. **b** Heat map revealing the expression profles of 419 DEGs. The colors depict high (red) and low (green) level of gene expression. The color alteration from green to black to red represents increasing expression. *DEMs* differentially expressed miRNAs, *DEGs* differentially expressed genes

Using the GSE90603 dataset, 59 DEMs were identified in the GBM samples compared with the peritumoral tissues, of which 37 were upregulated and 22 were downregulated (Additional file 1). The 20 DEMs with the lowest *p*-value are presented in Table 1. In total, 419 DEGs were obtained in the GSE90598 dataset, and among these, 77 were upregulated and 342 were downregulated (Additional file 2). The top 20 DEGs with the lowest *p*-value are presented in Table 2. Heatmaps of DEMs and DEGs are shown in Fig. 2.

**Table 1:** Top 20 DEMs between GBM sample compared with normal brain tissues

ID	logFC	adj.P.Val
hsa-miR-320d	2.226057281	4.84E-07
hsa-miR-455-3p	2.521827549	4.84E-07
hsa-miR-25-3p	3.144720167	5.08E-07
hsa-miR-500a-5p	2.346944027	9.88E-07
hsa-miR-28-3p	2.748360285	1.68E-06
hsa-miR-106b-3p	2.745815992	2.73E-06
hsa-miR-362-5p	2.202213072	2.79E-06
hsa-miR-155-5p	3.886573305	4.04E-06
hsa-miR-490-5p	− 2.531780737	5.53E-06
hsa-miR-21-5p	4.138010995	7.73E-06
hsa-miR-338-3p	− 2.728425739	7.73E-06
hsa-miR-424-3p	2.83234903	1.17E-05
hsa-miR-23a-3p	2.602961549	1.24E-05
hsa-miR-18a-5p	2.111435648	1.52E-05
hsa-miR-339-5p	2.623198868	1.97E-05
hsa-miR-15b-5p	3.178531266	1.97E-05
hsa-miR-6872-3p	2.196366981	2.41E-05
hsa-miR-93-5p	2.182576627	2.75E-05
hsa-mir-92b	2.64699354	3.27E-05
hsa-miR-181a-2-3p	2.532050498	3.60E-05

*DEMs* differentially expressed miRNAs, *GBM* glioblastoma

**Table 2:** Top 20 DEGs between GBM sample compared with normal brain tissues

ID	logFC	adj.P.Val
SEC14L5	− 2.410823841	2.49E-09
KCNT1	− 2.989158475	2.49E-09
PEX5L	− 3.806308716	2.33E-08
LGI3	− 3.224367902	3.81E-08
OPALIN	− 4.148538323	3.81E-08
CORO6	− 2.317401319	3.81E-08
GABRG1	− 4.377954642	4.80E-08
KCNH3	− 2.635371133	6.24E-08
ROGDI	− 2.067007874	1.02E-07
GRM3	− 3.433516194	1.08E-07
UNC13C	− 4.564034026	1.08E-07
RASGRF1	− 2.865726351	1.12E-07
CABP1	− 3.404983957	1.25E-07
PXDN	2.869541549	1.79E-07
SH3GL3	− 3.060067098	2.41E-07
PPP2R2C	− 2.912293455	2.57E-07
RAPGEF5	− 2.685003832	2.85E-07
RPH3A	− 3.228432626	2.87E-07
NECAB1	− 4.029953556	4.08E-07
ANK3	− 2.985792673	4.73E-07

*DEGs* differentially expressed genes, *GBM* glioblastoma

### Transcription factor (TF) and gene ontology (GO) analyses of DEMs

**Fig. 3:**
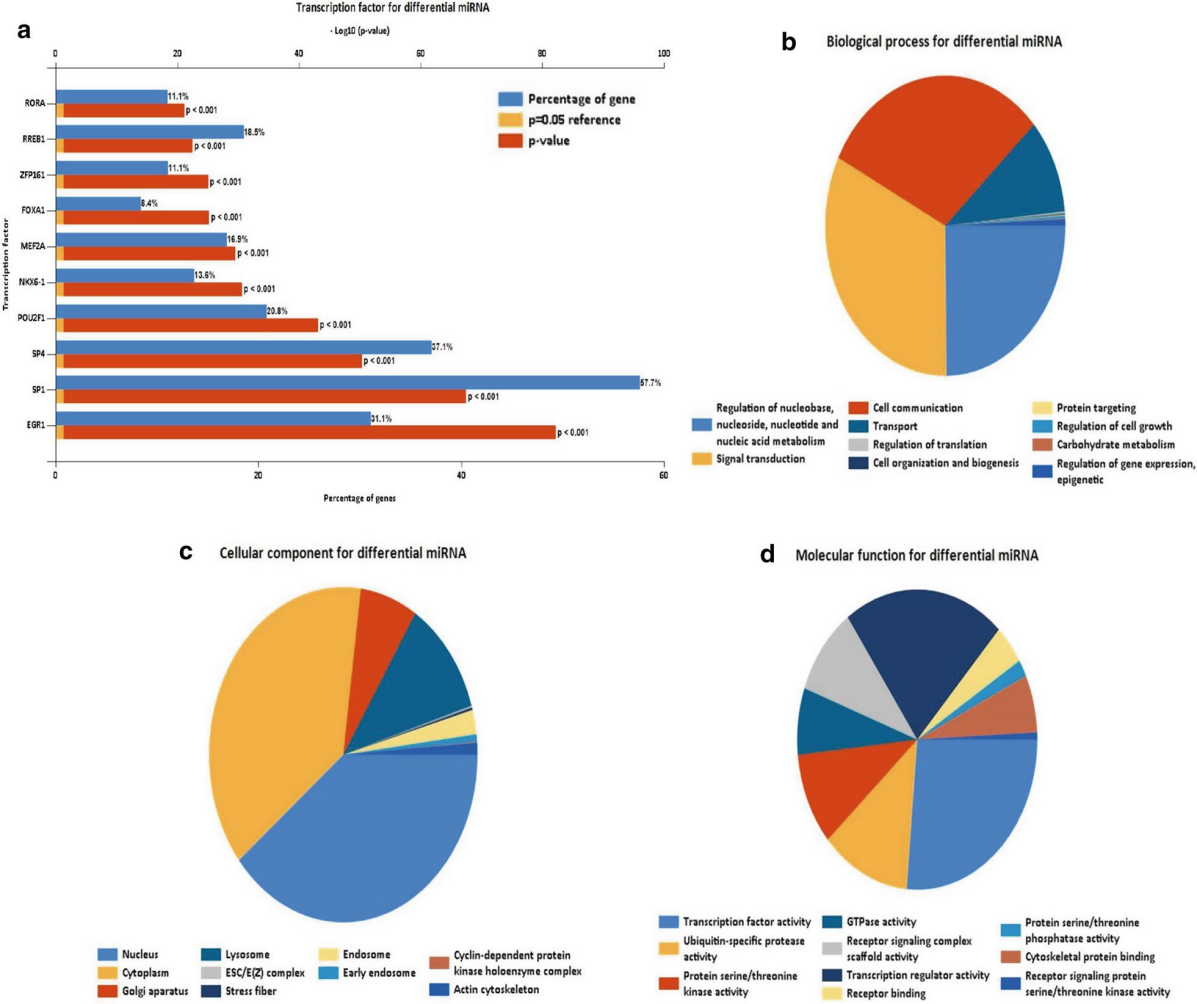
Top 10 TFs and GO analysis of DEMs associated with GBM. **a** Top 10 TFs of DEMs; **b** Top 10 BP of DEMs; **c** Top 10 CC of DEMs; **d** Top 10 MF of DEMs. TFs, transcription factors; *GO* Gene ontology, *DEGs* differentially expressed genes, *BP* biological processes, *CC* cellular component, *MF* molecular function

TF and GO analyses of DEMs were performed by FunRich software. The top ten most commonly expressed TFs associated with DEMS were *RORA*, *RREB1*, *ZFP161, FOXA1, MEF2A, NKX6-1, POU2F1, SP4, SP1,* and *EGR1* (Fig. 3a). GO enrichment analysis of the top ten DEMs was also performed using FunRich software. The result revealed that the majority of DEMs in the biological process (BP) category were enriched in “signal transduction”, “cell communication”, “nucleoside, nucleotide and nucleic acid metabolism”, and “transport” (Fig. 3b and Additional file 3). The GO cellular component (CC) enrichment analysis revealed that the majority of DEMs in this category were enriched in “nucleus”, “cytoplasm”, “lysosome”, and “golgi apparatus” (Fig. 3c and Additional file 4). In the molecular function (MF) category, the majority of DEMs were associated with “transcription factor activity”, “transcription regulator activity”, “protein serine/threonine kinase activity”, “ubiquitin-specific protease activity”, “receptor signaling, and complex scaffold activity” (Fig. 3d and Additional file 5).

### DEMs target gene prediction

The potential target genes (PTGs) of each DEM were obtained by using FunRich software. All 59 DEMs were selected for PTG prediction and 34 DEMs were identified. The FunRich tool generated 5147 PTGs for 34 DEMs. We then took the overlapping gene intersection of PTGs and DEGs, and analyzed the correlation of downregulated miRNAs with upregulated genes, upregulated miRNAs, and downregulated genes. There were nine core DEMs and 129 overlapping genes with a targeted relationship (Table 3).

**Table 3:** Overlapping genes between DEGs and DEMs target genes

miRNA	mRNA	Target	mirnaLogFC	mrnaLogFC
hsa-miR-338-3p	MYT1L	Target	− 2.728425739	3.946893602
hsa-miR-338-3p	RGS7BP	Target	− 2.728425739	4.314153573
hsa-miR-137	ANO4	Target	− 2.997290651	2.895129398
hsa-miR-137	ATP1B1	Target	− 2.997290651	6.271266512
hsa-miR-137	C11orf87	Target	− 2.997290651	3.557787858
hsa-miR-137	CEND1	Target	− 2.997290651	4.380926671
hsa-miR-137	CHGA	Target	− 2.997290651	3.254643461
hsa-miR-137	DIRAS2	Target	− 2.997290651	4.112396985
hsa-miR-137	EDIL3	Target	− 2.997290651	5.764240871
hsa-miR-137	EZH2	Target	− 2.997290651	4.634117944
hsa-miR-137	GABRA1	Target	− 2.997290651	2.537054605
hsa-miR-137	GRIN2A	Target	− 2.997290651	3.050424617
hsa-miR-137	HLF	Target	− 2.997290651	5.341249022
hsa-miR-137	NETO1	Target	− 2.997290651	4.134785091
hsa-miR-137	NRXN1	Target	− 2.997290651	5.784845966
hsa-miR-137	NRXN3	Target	− 2.997290651	3.652567346
hsa-miR-137	PLCB1	Target	− 2.997290651	5.750675061
hsa-miR-137	PTGFRN	Target	− 2.997290651	5.222125013
hsa-miR-137	PTPN5	Target	− 2.997290651	3.471111043
hsa-miR-137	RAPGEF5	Target	− 2.997290651	4.030838556
hsa-miR-137	RCAN2	Target	− 2.997290651	5.450737004
hsa-miR-137	RGS7BP	Target	− 2.997290651	4.314153573
hsa-miR-137	ST18	Target	− 2.997290651	3.050911173
hsa-miR-137	SYT1	Target	− 2.997290651	4.165893064
hsa-miR-137	TNC	Target	− 2.997290651	6.174176272
hsa-miR-137	UNC79	Target	− 2.997290651	4.330400236
hsa-miR-137	WIF1	Target	− 2.997290651	3.01009045
hsa-miR-128-3p	CABP1	Target	− 2.521510423	3.190706419
hsa-miR-128-3p	DIRAS2	Target	− 2.521510423	4.112396985
hsa-miR-128-3p	ERC2	Target	− 2.521510423	4.094505701
hsa-miR-128-3p	JAG1	Target	− 2.521510423	6.666037367
hsa-miR-128-3p	KLHDC8A	Target	− 2.521510423	5.045962448
hsa-miR-128-3p	PPFIA2	Target	− 2.521510423	4.623994699
hsa-miR-128-3p	PTPN5	Target	− 2.521510423	3.471111043
hsa-miR-128-3p	SAMD9L	Target	− 2.521510423	5.284575791
hsa-miR-128-3p	SNAP25	Target	− 2.521510423	6.690762972
hsa-miR-128-3p	UGT8	Target	− 2.521510423	5.440745657
hsa-miR-128-3p	UNC13C	Target	− 2.521510423	3.409978716
hsa-miR-218-5p	CELF4	Target	− 2.721703277	3.962404699
hsa-miR-218-5p	DIRAS2	Target	− 2.721703277	4.112396985
hsa-miR-218-5p	DLG2	Target	− 2.721703277	4.123750776
hsa-miR-218-5p	ERC2	Target	− 2.721703277	4.094505701
hsa-miR-218-5p	GABRB2	Target	− 2.721703277	3.053748336
hsa-miR-218-5p	GNAO1	Target	− 2.721703277	7.321852005
hsa-miR-218-5p	GNG3	Target	− 2.721703277	5.769726039
hsa-miR-218-5p	GRM3	Target	− 2.721703277	4.486725834
hsa-miR-218-5p	HLF	Target	− 2.721703277	5.341249022
hsa-miR-218-5p	HS6ST3	Target	− 2.721703277	2.808269582
hsa-miR-218-5p	KCNT1	Target	− 2.721703277	3.426516123
hsa-miR-218-5p	KLHDC8A	Target	− 2.721703277	5.045962448
hsa-miR-218-5p	MYT1L	Target	− 2.721703277	3.946893602
hsa-miR-218-5p	NECAB1	Target	− 2.721703277	4.155553563
hsa-miR-218-5p	NRXN1	Target	− 2.721703277	5.784845966
hsa-miR-218-5p	NRXN3	Target	− 2.721703277	3.652567346
hsa-miR-218-5p	PEX5L	Target	− 2.721703277	3.759139098
hsa-miR-218-5p	PPP2R2C	Target	− 2.721703277	3.149976347
hsa-miR-218-5p	RAP1GAP	Target	− 2.721703277	4.163641598
hsa-miR-218-5p	RAPGEF4	Target	− 2.721703277	5.748455022
hsa-miR-218-5p	SCN2B	Target	− 2.721703277	3.361364656
hsa-miR-218-5p	SERPINI1	Target	− 2.721703277	3.748145315
hsa-miR-218-5p	STXBP1	Target	− 2.721703277	8.328766888
hsa-miR-218-5p	TNC	Target	− 2.721703277	6.174176272
hsa-miR-218-5p	UGT8	Target	− 2.721703277	5.440745657
hsa-miR-7-5p	ATP2B2	Target	− 3.075240145	4.586872611
hsa-miR-7-5p	GABRA1	Target	− 3.075240145	2.537054605
hsa-miR-7-5p	HCN1	Target	− 3.075240145	4.045514621
hsa-miR-7-5p	HPCAL4	Target	− 3.075240145	3.717500854
hsa-miR-7-5p	NECAB1	Target	− 3.075240145	4.155553563
hsa-miR-7-5p	RAB11FIP4	Target	− 3.075240145	4.429233935
hsa-miR-7-5p	RBFOX3	Target	− 3.075240145	3.734457069
hsa-miR-7-5p	RGS7BP	Target	− 3.075240145	4.314153573
hsa-miR-7-5p	SNCA	Target	− 3.075240145	3.750171197
hsa-miR-139-5p	GABRA1	Target	− 2.750606611	2.537054605
hsa-miR-124-3p	ANO5	Target	− 3.819939565	3.263031615
hsa-miR-124-3p	ASPA	Target	− 3.819939565	3.843028446
hsa-miR-124-3p	BSN	Target	− 3.819939565	4.266708475
hsa-miR-124-3p	C11orf87	Target	− 3.819939565	3.557787858
hsa-miR-124-3p	C1QL3	Target	− 3.819939565	3.264695246
hsa-miR-124-3p	CADM2	Target	− 3.819939565	5.581287413
hsa-miR-124-3p	CBLN2	Target	− 3.819939565	2.86492923
hsa-miR-124-3p	CCL2	Target	− 3.819939565	5.53611632
hsa-miR-124-3p	CDH9	Target	− 3.819939565	2.597421507
hsa-miR-124-3p	CNTN1	Target	− 3.819939565	5.503812542
hsa-miR-124-3p	COL4A1	Target	− 3.819939565	4.774842046
hsa-miR-124-3p	DLGAP2	Target	− 3.819939565	2.690594307
hsa-miR-124-3p	EZH2	Target	− 3.819939565	4.634117944
hsa-miR-124-3p	FAM171A1	Target	− 3.819939565	5.410041936
hsa-miR-124-3p	FRRS1L	Target	− 3.819939565	4.044676543
hsa-miR-124-3p	JAG1	Target	− 3.819939565	6.666037367
hsa-miR-124-3p	JAKMIP1	Target	− 3.819939565	3.090572083
hsa-miR-124-3p	KCNA1	Target	− 3.819939565	3.007882911
hsa-miR-124-3p	KCNJ3	Target	− 3.819939565	3.130437439
hsa-miR-124-3p	KIF5A	Target	− 3.819939565	6.64331389
hsa-miR-124-3p	LAMC1	Target	− 3.819939565	5.46622199
hsa-miR-124-3p	LRRC7	Target	− 3.819939565	3.916089849
hsa-miR-124-3p	MAP7	Target	− 3.819939565	5.190958174
hsa-miR-124-3p	NAMPT	Target	− 3.819939565	4.854083944
hsa-miR-124-3p	NEFM	Target	− 3.819939565	3.574096381
hsa-miR-124-3p	NEGR1	Target	− 3.819939565	4.141134838
hsa-miR-124-3p	NID1	Target	− 3.819939565	5.602483311
hsa-miR-124-3p	NKAIN2	Target	− 3.819939565	4.323047449
hsa-miR-124-3p	PALM	Target	− 3.819939565	5.25214283
hsa-miR-124-3p	PLCB1	Target	− 3.819939565	5.750675061
hsa-miR-124-3p	PTGFRN	Target	− 3.819939565	5.222125013
hsa-miR-124-3p	SLC7A14	Target	− 3.819939565	3.168925912
hsa-miR-124-3p	STEAP3	Target	− 3.819939565	4.951991733
hsa-miR-124-3p	TBR1	Target	− 3.819939565	2.713595548
hsa-miR-124-3p	UGT8	Target	− 3.819939565	5.440745657
hsa-miR-124-3p	VCAN	Target	− 3.819939565	6.729783912
hsa-miR-383-5p	MAL2	Target	− 3.452155995	2.662950301
hsa-miR-383-5p	PPFIA2	Target	− 3.452155995	4.623994699
hsa-miR-383-5p	VEGFA	Target	− 3.452155995	6.507050912
hsa-miR-138-5p	DGKE	Target	− 2.820786706	2.844643402
hsa-miR-138-5p	EZH2	Target	− 2.820786706	4.634117944
hsa-miR-138-5p	HS6ST3	Target	− 2.820786706	2.808269582
hsa-miR-138-5p	MGAT5B	Target	− 2.820786706	3.750177501
hsa-miR-138-5p	NETO1	Target	− 2.820786706	4.134785091
hsa-miR-138-5p	NPTX1	Target	− 2.820786706	3.918195841
hsa-miR-138-5p	PLLP	Target	− 2.820786706	4.91045837
hsa-miR-138-5p	PTGFRN	Target	− 2.820786706	5.222125013
hsa-miR-138-5p	RCAN2	Target	− 2.820786706	5.450737004
hsa-miR-138-5p	RIMS2	Target	− 2.820786706	3.387452792
hsa-miR-138-5p	SCN3B	Target	− 2.820786706	4.019176399
hsa-miR-138-5p	SH3GL2	Target	− 2.820786706	3.166496716
hsa-miR-138-5p	SLC17A7	Target	− 2.820786706	4.686584977
hsa-miR-138-5p	SLC6A17	Target	− 2.820786706	4.443401567
hsa-miR-138-5p	SNAP25	Target	− 2.820786706	6.690762972
hsa-miR-138-5p	SRRM4	Target	− 2.820786706	3.659380613
hsa-miR-138-5p	WEE1	Target	− 2.820786706	4.41487692

*DEMs* differentially expressed miRNAs, *DEGs* differentially expressed genes

### MiRNA-gene regulatory network construction

**Fig. 4:**
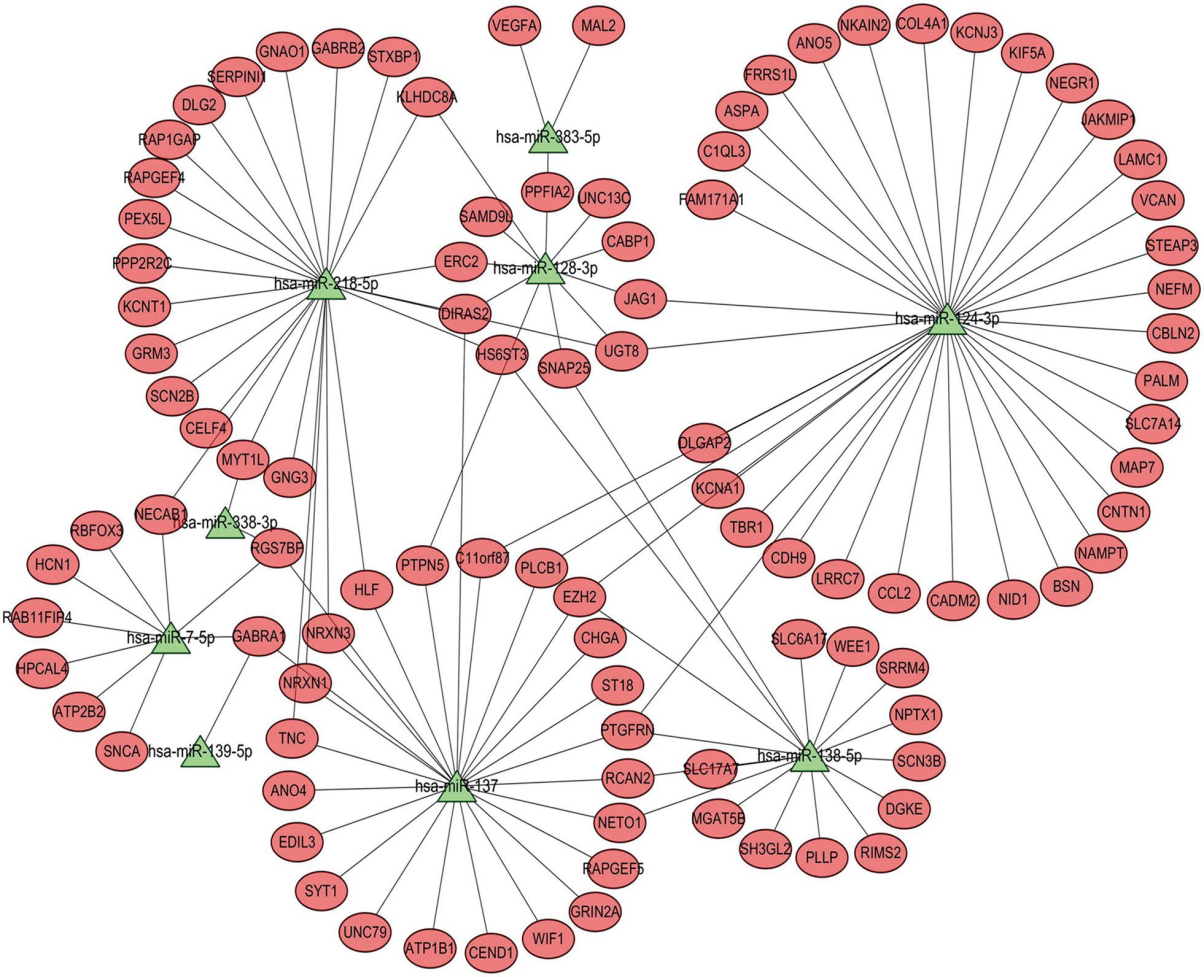
The networks of validated DEMs and overlapping genes between DEGs and DEMs target genes. Red nodes represent upregulated factors, while green nodes represent downregulated factors. Triangles represent DEMs, and circles represent overlapping genes. *DEMs* differentially expressed miRNAs, *DEGs* differentially expressed genes

According to the information obtained from the nine core DEMs and 129 overlapping target genes, core DEMs and target gene regulatory networks were constructed and visualized using Cytoscape software (Fig. 4). Overall, the data indicate that the nine core DEMs and 129 overlapping target genes may play important roles in the diagnosis and prognosis of glioma.

### GO and Kyoto Encyclopedia of Genes and Genomes(KEGG) pathway enrichment analysis of overlapping target genes

**Fig. 5:**
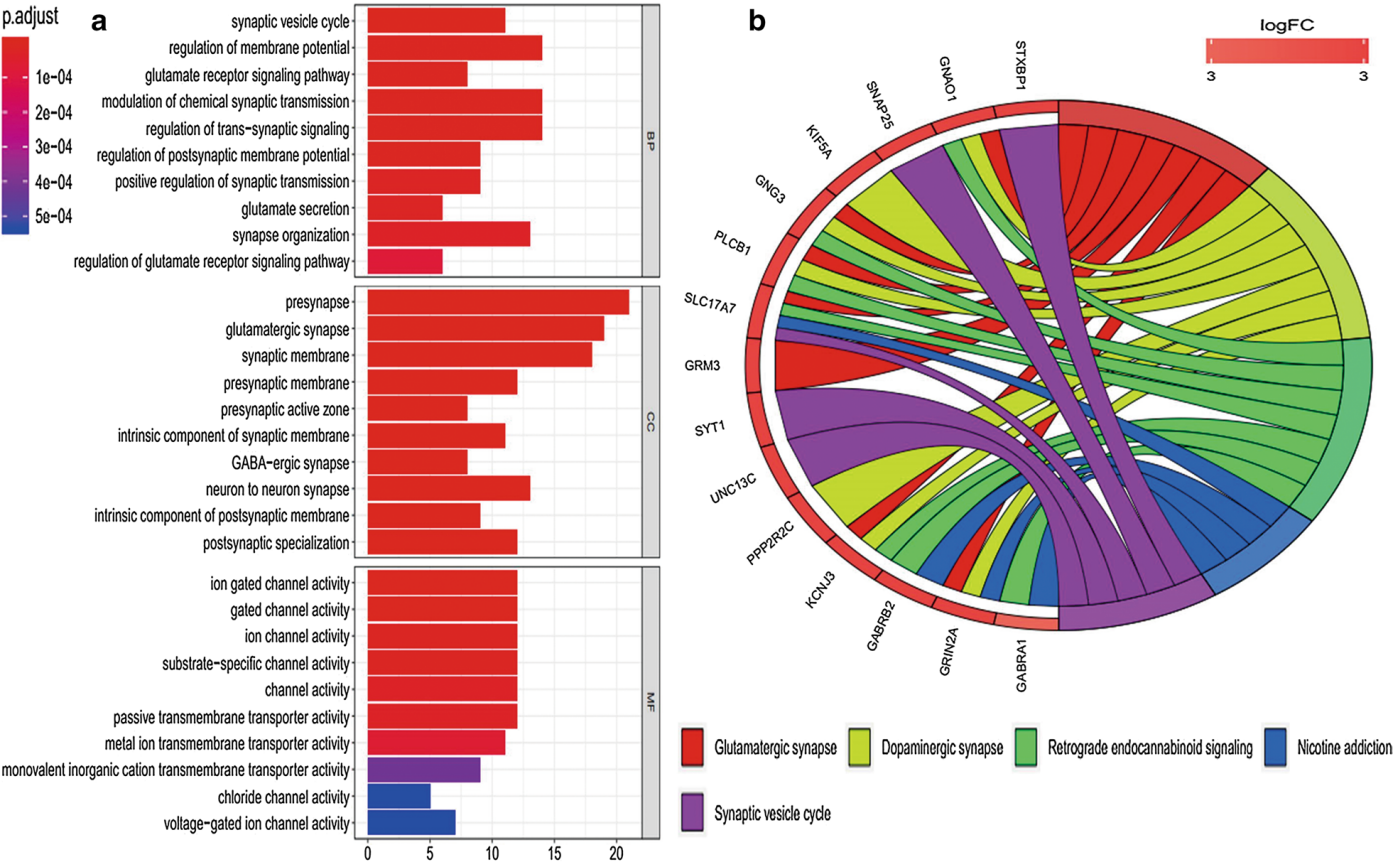
GO and KEGG analysis of overlapping genes between DEGs and DEMs target genes. **a** GO analysis; **b** KEGG analysis. *DEMs* differentially expressed miRNAs, *DEGs* differentially expressed genes, *GO* Gene ontology, *KEGG* Kyoto Encyclopedia of Genes and Genomes

To explore roles that overlapping target genes paly in biological functions, GO and KEGG pathway analysis were performed. The overlapping genes were significantly enriched in BP, including “synaptic vesicle cycle”, “regulation of membrane potential”, “glutamate receptor signaling pathway”, “modulation of chemical synaptic transmission” and so on (Fig. 5a and Additional file 6). For MF, The overlapping target genes were mainly related to “ion gate channel activity”, “gated channel activity”, “ion channel activity”, “substrate-specific channel activity” and so on (Fig. 5a and Additional file 6). In addition, CC analysis indicated that the overlapping target genes were involved in “presynapse”, “glutamatergic synapse”, “synaptic membrane”, “presynaptic membrane” and so on (Fig. 5a Additional file 6). KEGG analysis revealed that the overlapping target genes were mostly enriched in “glutamatergic synapse”, “dopaminergic synapse”, “retrograde endocannabinoid signaling”, “nicotine addiction” and “synaptic vesicle cycle” (Fig. 5b and Additional file 7).

### Survival analysis

**Fig. 6:**
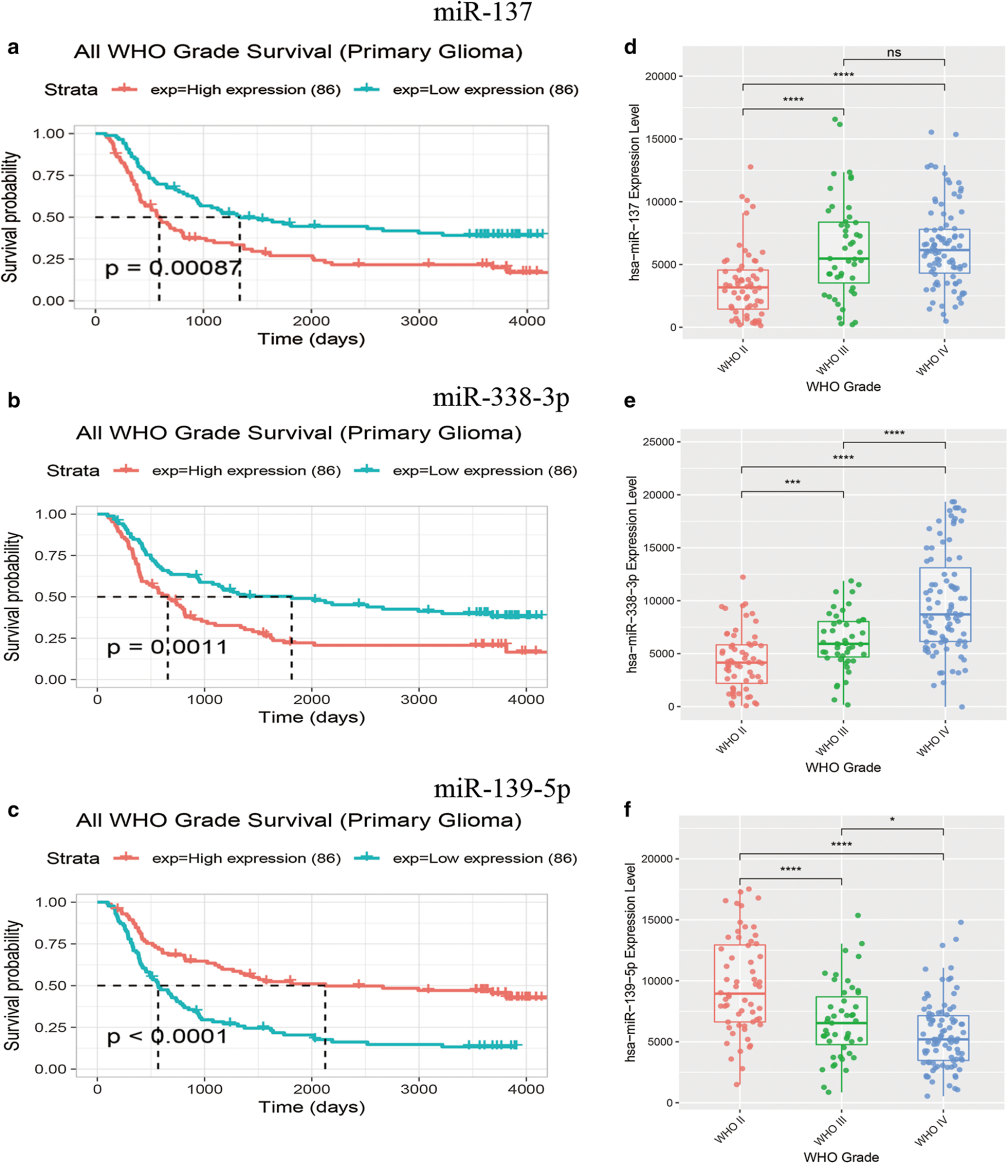
The prognostic value of three DEMs in GBM and all grades glioma. The overall survival rate **a** Prognostic value of miR-137 in all grades glioma; **b** Prognostic value of miR-338-3p in all grades glioma; **c** Prognostic value of miR-139-5p in all grades glioma; **d–e **The expression of miR-137 and miR-338-3p was positively correlated with glioma grade. **f** the expression of miR-139-5p was negatively correlated with glioma grade

The relationship between the expression of the nine core DEMs (miR-338-3p, miR-137, miR-128-3p, miR-218-5p, miR-7-5p, miR-139-5p, miR-124-3p, miR-383-5p, and miR-138-5p) and the overall survival of patients with all grades of glioma was analyzed using the Chinese Glioma Genome Atlas (CGGA). Only three miRNA clinical datasets existed in the CGGA database (miR-137, miR-139-5p, and miR-338-3p). We found that the high expression of miR-137 and miR-338-3p were associated with poor overall survival rate in patients with all grades of glioma (Fig. 6a, b; *p* < 0.01) while low expression of miR-139-5p was associated with poor overall survival rates (Fig. 6c; *p* < 0.01).

To reveal the correlation of core miRNA expression profiles with glioma grade, we compared core miRNAs distribution data of grade II–IV cases obtained in the CGGA database. The expression of miR-137 and miR-338-3p was positively correlated with glioma grade (Fig. 6d, e), whereas the expression of miR-139-5p was negatively correlated with glioma grade (Fig. 6f).

### GABRA1 as the functional target of miR-139-5p

**Fig. 7:**
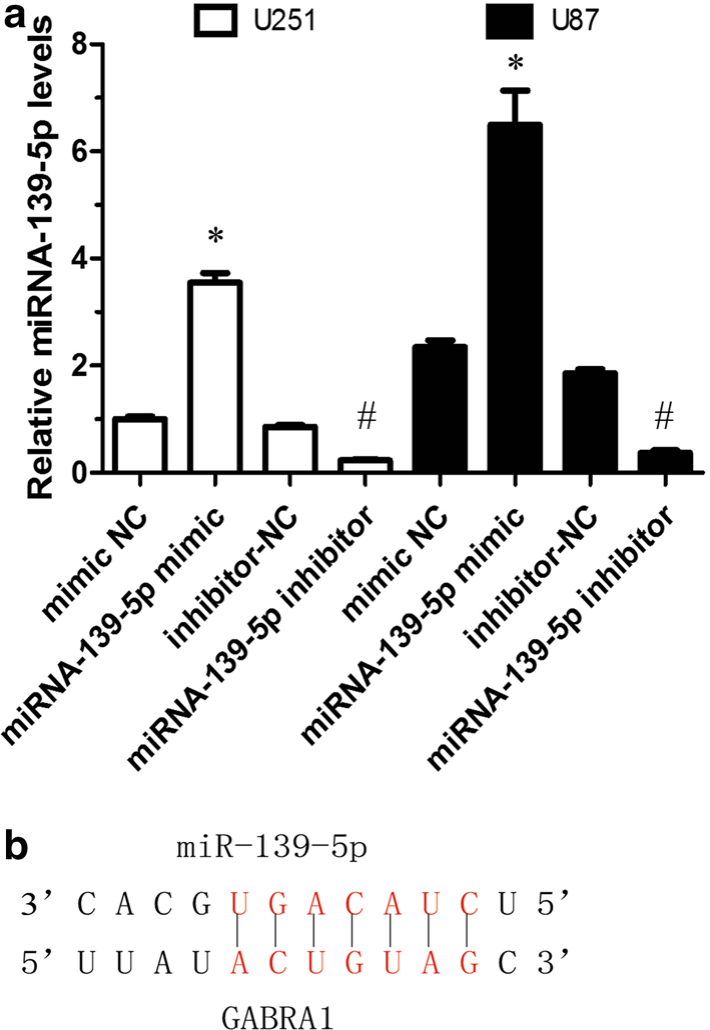
**a** qRT-PCR analysis of GABRA1 mRNA expression in U251 and U87 cells transfected with miR-139-5p mimic or miR-139-5p inhibitor. **b **Putative miR-139-5p binding sites in the 3′-UTR sequence of GABRA1 genes. *p < 0.05, vs mimic NC; #p < 0.05 vs inhibitor-NC

Combining the literature search and the bioinformatics analyses, miR-139-5p and its potential target, GABRA1, were selected for further analysis. We next aimed to ascertain the underlying molecular mechanisms by which miR-139-5p exerted its tumor suppressing roles in glioma. Hence, an miR-139-5p mimic and inhibitor were respectively transfected into the U251 and U87 cell lines. The efficacy of transfection was determined by RT-PCR, with results indicating that the relative expression of miR-139-5p was significantly increased in U251 and U87 cells after transfection of the miR-139-5p mimic (Fig. 7a). Conversely, miR-139-5p expression was dramatically downregulated after transfection with the miR-139-5p inhibitor (Fig. 7a).

**Fig. 8:**
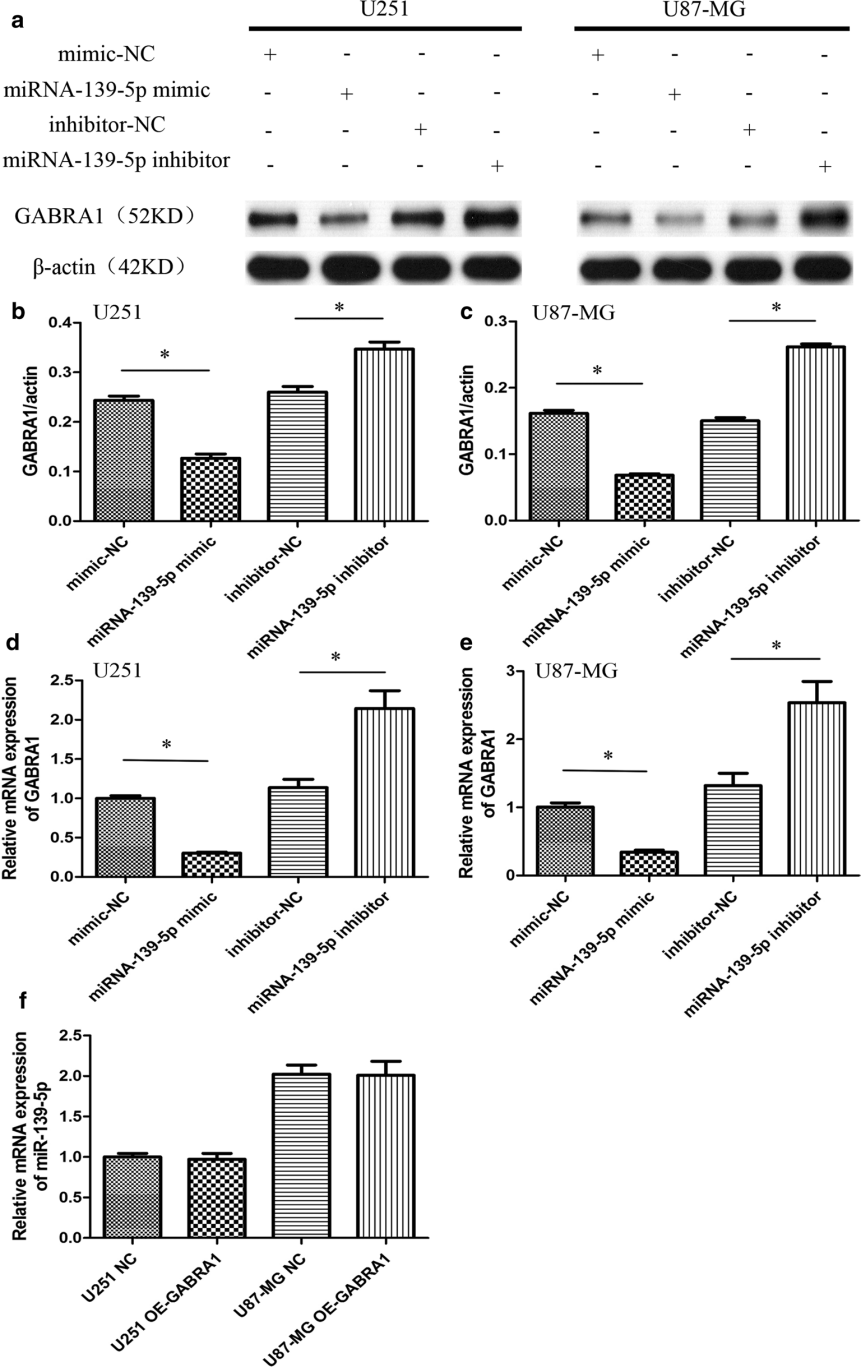
**a–c** Western blot assay of GABRA1 protein levels in U251 and U87 cells transfected with miR-139-5p mimic or miR-139-5p inhibitor. **d**-**e** qRT-PCR analysis of GABRA1 mRNA levels in U251 and U87 cells transfected with miR-139-5p mimic or miR-139-5p inhibitor. **f** qRT-PCR analysis of miR-139-5p mRNA levels in U251 and U87 cells transfected with GABRA1 or control. *p < 0.05

Both the previous bioinformatics analyses and online miRNA database analysis (Target Scan) indicated that GABRA1 could be a target of miR-139-5p, according to the putative target sequence in the GABRA1 3’Untranslated Regions (UTR) (Fig. 7b). RT-PCR and western blot analyses were carried out to further identify whether GABRA1 expression was indeed regulated by miR-139-5p. The results indicated that the mRNA and protein levels of GABRA1 were significantly increased after transfection with the miR-139-5p inhibitor, while the levels were reduced after transfection with the miR-139-5p mimics, in U251 and U87 cells (Fig. 8a–e). In addition, the results of RT-PCR assays indicated that the over-expression of GABRA1 had no effect on the expression of miR-139-5p (Fig. 8f). These results suggested that miR-139-5p could directly target GABRA1 and negatively regulate its expression.

### MiR-139-5p regulates glioma cell migration and invasion through targeting of GABRA1.

**Fig. 9:**
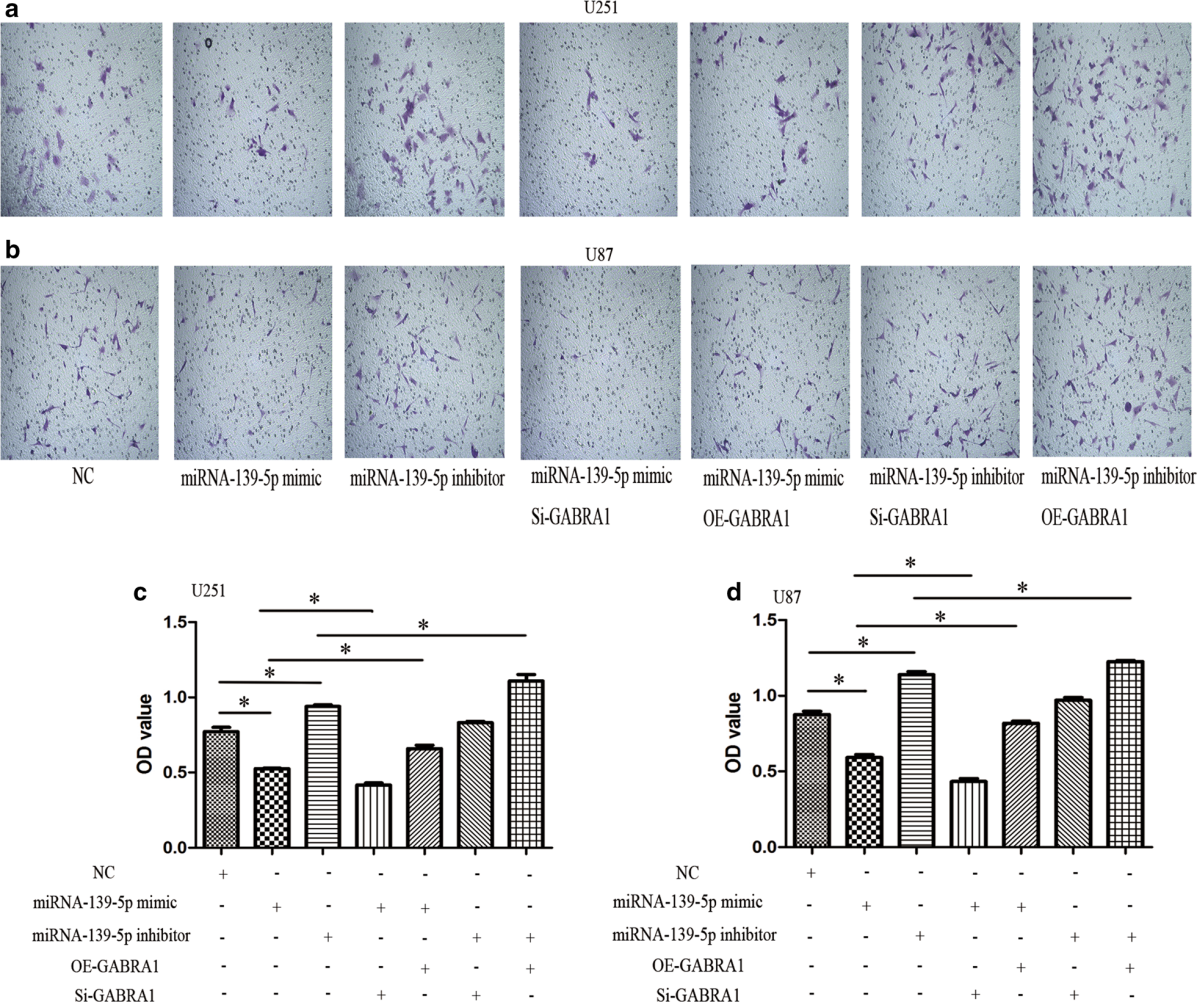
Transwell assay in U251 and U87 cells after transfection with miRNA-139-5p mimic or miRNA-139-5p inhibitor and quantification of relative number of invaded cells. Each assay was repeated at least three times. *P < 0.05

**Fig. 10:**
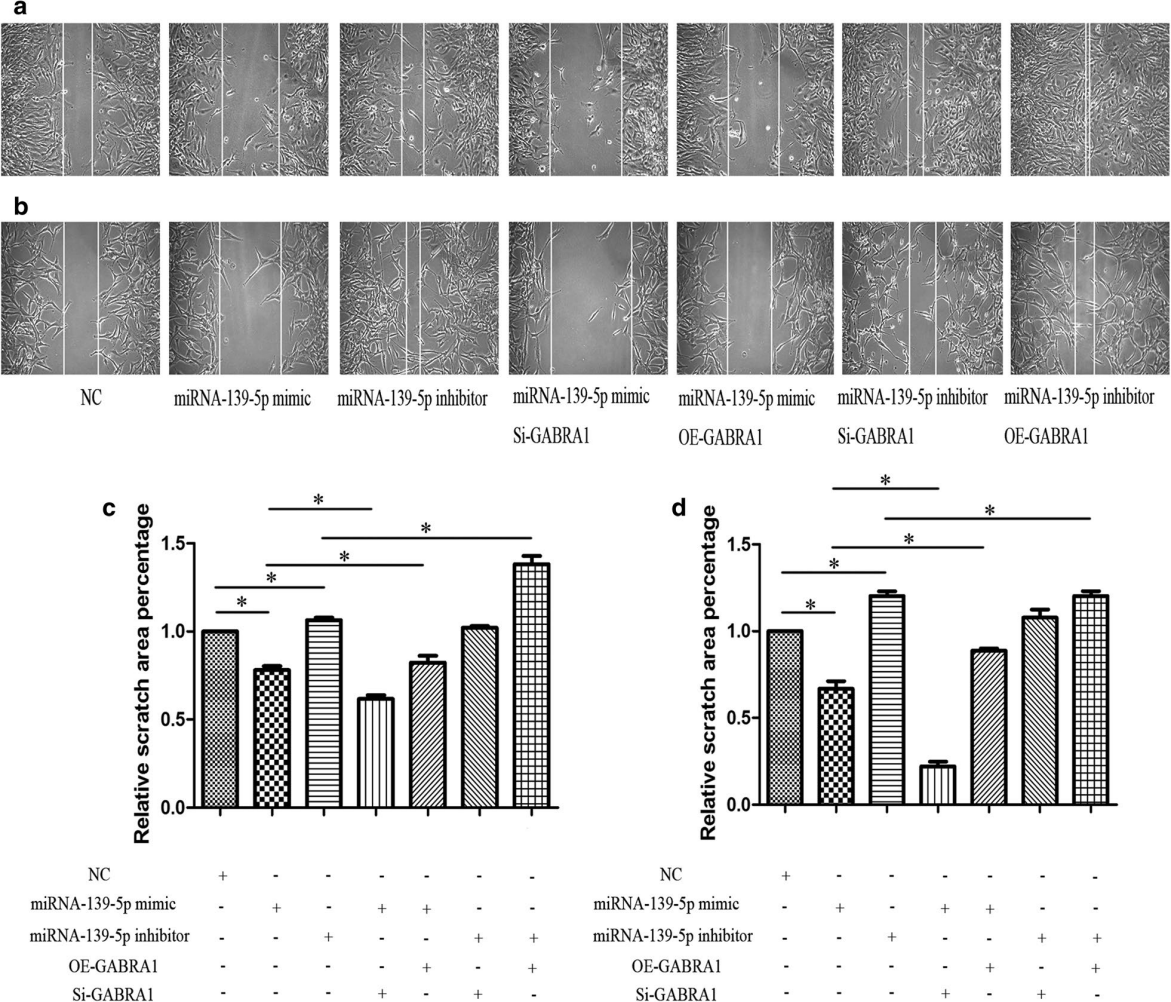
Wounded healing assay in U251 and U87 cells after transfection with miRNA-139-5p mimic or miRNA-139-5p inhibitor and quantification of relative scratch area. Each assay was repeated at least three times. *P < 0.05

We next performed transwell assays and wound healing assays to evaluate the effects of miR-139-3p on the migratory and invasive capabilities of U251 and U87 cells. Both transwell and wound healing assays indicated that miR-139-5p significantly repressed U251 and U87 cell migration and invasion (Figs. 9 and 10). Loss-of-function experiments suggested that the miR-139-5p inhibitor elevated U251 and U87 cell migration and invasion (Figs. 9 and 10).

Next, we explored whether GABRA1 mediated the effects of miR-139-5p on the cell migration and invasion of U251 and U87 cells. Specifically, rescue experiments were carried out by co-transfecting the miR-139-5p mimic/inhibitor with or without GABRA1 overexpression (OE) and silencing (Si). The miR-139-5p inhibitor resulted in a significant increase in the migration and invasion, while miR-139-5p mimic resulted in decreased migration and invasion, of glioma cells. OE-GABRA1 remarkably rescued the suppressive effects of the miR-139-5p mimic on the invasive abilities of U251 and U87 cells (Figs. 9 and 10). In addition, cotransfection of the miR-139-5p inhibitor and OE-GABRA1 notably promoted migration and invasion compared with transfection of the miR-139-5p inhibitor alone. Cotransfection of the miR-139-5p inhibitor and Si-GABRA1 significantly inhibited the migration and invasion of U251 and U87 cells (Figs. 9 and 10). Taken together, our data indicated that miR-139-5p modulates glioma cell migration and invasion by targeting GABRA1.

### MiR-139-5p regulates glioma cell proliferation and apoptosis through targeting of GABRA1

**Fig. 11:**
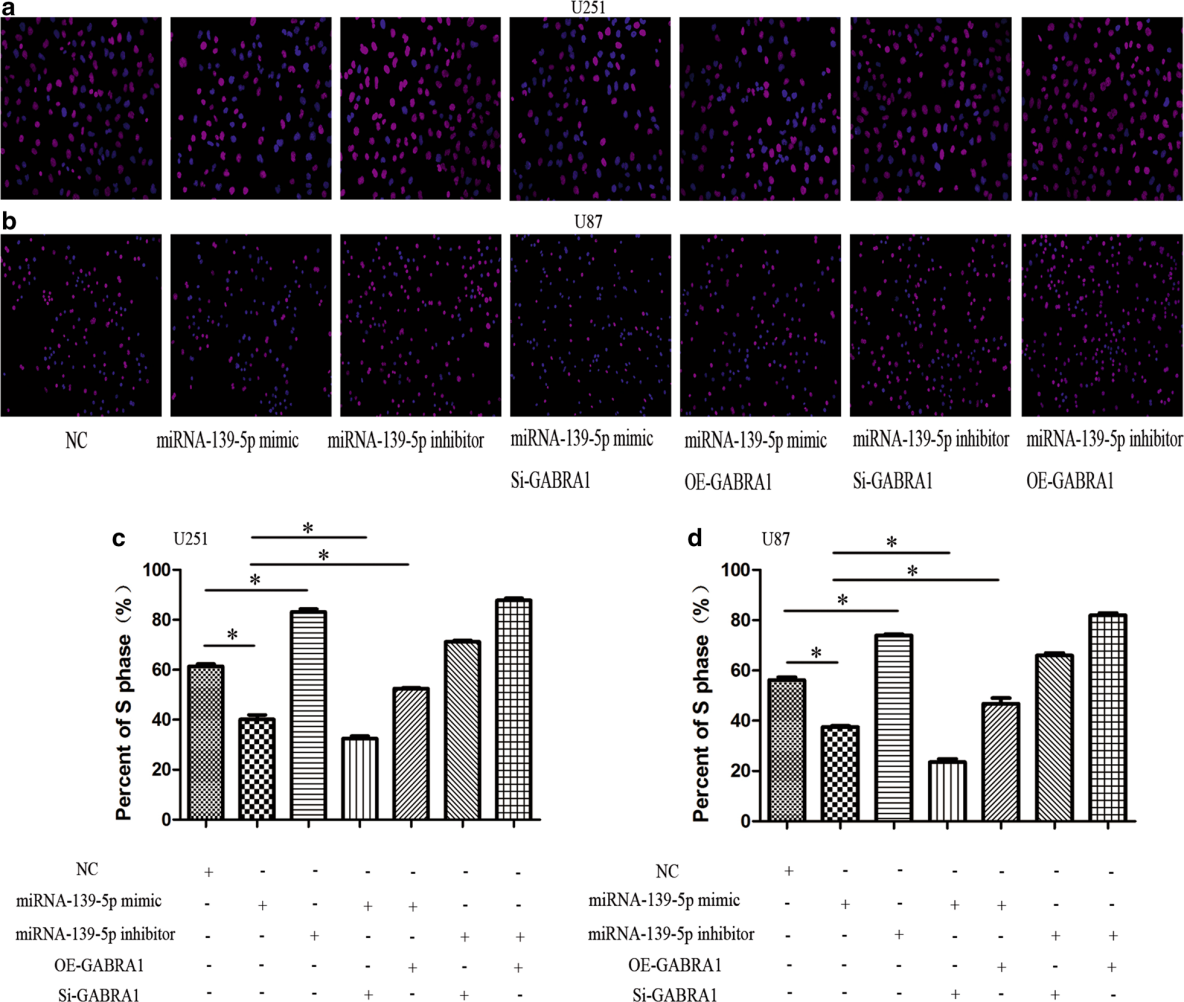
EdU assay in U251 and U87 cells after transfection with miRNA-139-5p mimic or miRNA-139-5p inhibitor and quantification of S phase. Each assay was repeated at least three times. *P < 0.05

**Fig. 12:**
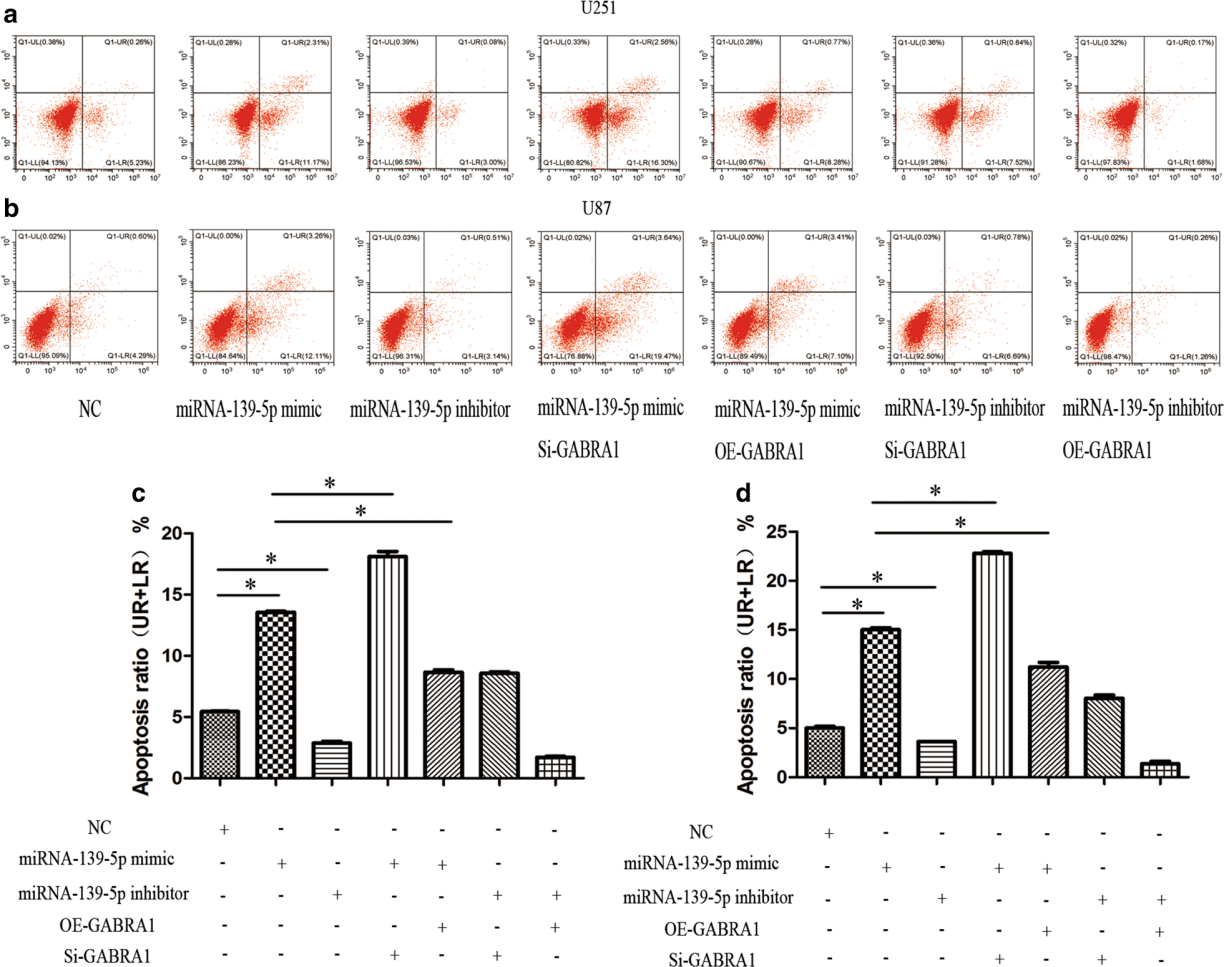
Flow cytometry assay in U251 and U87 cells after transfection with miRNA-139-5p mimic or miRNA-139-5p inhibitor and quantification of apoptosis ratio. Each assay was repeated at least three times. *P < 0.05

Cell proliferation and apoptosis experiments were then performed in U251 and U87 cell lines. The results of the quantitative measurement of EdU-positive cells indicated that downregulation of miR-139-5p promoted the proliferation, while upregulation of miR-139-5p inhibited the proliferation, of U251 and U87 cells (Fig. 11). Flow cytometric analysis showed that the cell apoptosis rate was notably higher in the miR-139-5p mimic group while the rate was lower in the miR-139-5p inhibitor group compared with the NC group, indicating that miR-139-5p induced the apoptosis of U251 and U87 cells (Fig. 12).

Since GABRA1 is directly targeted by miR-139-5p, we speculated that GABRA1 mediates the function of miR-139-5p. To test this hypothesis, we overexpressed GABRA1 in the miR-139-5p mimic group and found that GABRA1 overexpression rescued the miR-139-5p-induced inhibition of proliferation and increase in apoptosis (Figs. 11 and 12). Cotransfection of the miR-139-5p mimic and Si-GABRA1 significantly suppressed proliferation and promoted apoptosis, compared with transfection of miR-139-5p mimic alone (Figs. 11 and 12). The results revealed that the restoration of GABRA1 markedly reversed miR-139-5p-mediated glioma inhibitory effects.

## Discussion

MiRNAs are a group of short and non-coding RNA molecules that influence the biological characteristics of GBM cells [8, 9]. Mounting evidence has shown that miRNAs can act as cancer-suppressing factors as well as oncogenes [10–12]. MiRNAs play important roles in the tumorigenesis and progression, invasion, and metastasis of GBM by upregulating or downregulating cancer-related gene expression levels, and, therefore, have become promising biomarkers in the diagnosis and prognosis glioma [13].

In this work, bioinformatics technology was used to identify candidate miRNA biomarkers of glioblastoma via analysis of microarray data. As a result, 59 DEMs and 419 DEGs were identified in the GBM samples compared with healthy brain tissues.

The prediction and GO enrichment analyses performed on the top ten DEMs showed that the majority of DEMs were enriched in signal transduction (BP) and transcription factor activity (MF), and were primarily located in the nucleus (CC). To explore the interactions between DEMs and their corresponding target genes, we performed GO enrichment and KEGG pathway analyses using the list of targeted genes. GO term analysis indicated that the target genes were mainly involved in synaptic vesicle cycle and regulation of membrane potential. KEGG analysis of the target genes identified that they were involved in synaptic relevant pathways, including the glutamatergic synapse, dopaminergic synapse, and synaptic vesicle cycle signaling pathways. The results also indicated that the synaptic related genes aide in the prognosis of patients with glioma, which can improve objectivity in clinical judgment.

Subsequently, the integration analysis of the miRNA-target gene regulatory pairs and DEGs revealed that 129 overlapping genes were regulated by nine DEMs. Further analysis revealed that miR-137, miR-139-5p, and miR-338-3p were associated with prognosis in patients with all grades of glioma.

MiRNA-137 is an miRNA that is widely expressed in the central nervous system, and is particularly specific to hippocampal tissue [14]. It has been reported that the biological function of miRNAs may be related to synaptic plasticity and transmission [15, 16]. Previous studies have indicated lower expression of miRNA-137 in tumor tissues, as compared with healthy tissues, in gastric cancer, colon cancer, and oral squamous cell carcinoma, suggesting that miRNA-137 acts as a suppressor [17, 18].

It has been reported that miR-338-3p functions as a tumor suppressor in several types of cancers [19–21]. Overexpression of miR-338-3p attenuated malignant biological behaviors of cells in gastric cancer and non-small cell lung cancer [19–21]. In the central nervous system, expression of miR-338-3p increases significantly as the dentate gyrus matures, and peaks in mature neurons [22]. Clinical data has also shown that low expression of miR-338-3p corresponds to a decrease in overall survival and disease-free survival [22].

Here, we found differing conclusions from previous research based on data from the CGGA. Lower expression of miRNA-137 and miR-338-3p was found in glioma tissue than in peritumoral tissue, suggesting that these two miRNAs may be tumor suppressor genes, consistent with previously reported in the literature. However, in the CGGA data, high expression of miRNA-137 and miR-338-3p was also associated with poor overall survival in glioma patients. The expression of miRNA-137 and miR-338-3p were positively correlated with glioma grade, suggesting that both miRNAs might be cancer-promoting factors.

MiR-139-5p is considered as a cancer suppressor because its expression level is downregulated in several types of cancer, such as prostate [23], pancreatic [24], hepatocellular carcinoma [25, 26] and non-small cell lung cancer [27]. In GBM, miR-139-5p may suppress tumor cell invasion and migration via targeting ZEB1 and ZEB2 [28]. In addition, miR-139 has been shown to suppress glioma cell proliferation and enhance temozolomide-induced apoptosis [29].

More recently, several in depth studies have shown there are biophysical interactions between glioma cells and neurons, and that neurons can activate related neurotransmitter receptors through the synaptic connection with glioma cells to promote the growth of glioma cells. [30, 31]. Gamma-aminobutyric acid(GABA), the most common inhibitory neurotransmitter in the brain, binds to the GABA-A channel. The GABA-A channel consists of subunit isoforms (two -subunits, two -subunits, and a third type of subunit), and is the most common type of GABA receptor. [32, 33]. The GABRA1 subunit is highly expressed in the central nervous system, as is one of the key subunits of GABA receptors. There are few studies on the relationship between GABRA1 expression and gliomas, and any that do exist have been controversial in regard to the expression level of GABRA1 in gliomas. D'Urso’s study indicated a lack of GABRA1 mRNA expression in glioblastoma primary cultures via northern blot and immunohistochemical analysis [34]. Another study showed that GABRA1 was expressed in gliomas, and that the expression level was highest in WHO grade II gliomas [35]. The effect of GABRA1 expression on the malignant biological behavior of glioma has not been reported. Available literature suggests that endogenous GABA receptor activity within glioma cells has a significant impact on tumor development [36]. Our experimental results in vitro suggest that GABRA1 is expressed in the glioma cell lines U251 and U87, and through overexpression and silencing experiments, we found that GABRA1 plays a role in promoting the growth of gliomas, which is directly regulated by miR-139-5p.

## Conclusions

In conclusion, we analyzed a combination of core miRNAs and target gene expressions between GBM and peritumoral tissue to further identify a set of promising diagnostic and prognostic biomarkers. We showed that miR-139-5p expression levels were decreased in glioma samples and that the miR-139-5p expression was significantly negatively correlated with prognosis of glioma patients. In addition, we discovered that miR-139-5p expression had a negative correlation with GABRA1 expression in U251 and U87 cells. Moreover, we found that miR-139-5p exerted tumor anti-tumor functions in glioma by directly targeting GABRA1.

## Supplementary Information

**Additional file 1: **All DEMs between GBM sample compared with normal brain tissues.

**Additional file 2**: All DEGs between GBM sample compared with normal brain tissues.

**Additional file 3**: GO enrichment of DEMs(BP)

**Additional file 4**: GO enrichment of DEMs(CC)

**Additional file 5**: GO enrichment of DEMs(MF)

**Additional file 6**: GO analysis of the overlapping target genes

**Additional file 7**: KEGG analysis of the overlapping target genes

## Data Availability

The datasets used or analyzed during the current study are available from The Chinese Glioma Genome Atlas (http://www.cgga.org.cn/).
